# *Trypanosoma cruzi*/Triatomine Interactions—A Review

**DOI:** 10.3390/pathogens14040392

**Published:** 2025-04-17

**Authors:** Günter A. Schaub

**Affiliations:** Zoology/Parasitology, Ruhr-University Bochum, Universitätsstr. 150, 44780 Bochum, Germany; guenter.schaub@rub.de

**Keywords:** antibacterial compounds, Chagas disease, interactions, microbiota, mutualistic symbionts, Triatominae, *Trypanosoma cruzi*

## Abstract

This review summarizes the interactions between *Trypanosoma cruzi*, the etiologic agent of Chagas disease, and its vectors, the triatomines, and highlights open questions. Four important facts should be emphasized at the outset: (1) The development of *T. cruzi* strains and their interactions with the mammalian host and the insect vector vary greatly. (2) Only about 10 of over 150 triatomine species have been studied for their interactions with the protozoan parasite. (3) The use of laboratory strains of triatomines makes generalizations difficult, as maintenance conditions influence the interactions. (4) The intestinal microbiota is involved in the interactions, but the mutualistic symbionts, Actinomycetales, have so far only been identified in four species of triatomines. The effects of the vector on *T. cruzi* are reflected in a different colonization ability of *T. cruzi* in different triatomine species. In addition, the conditions in the intestine lead to strong multiplication in the posterior midgut and rectum, with infectious metacyclic trypomastigotes developing almost exclusively in the latter. Starvation and feeding of the vector induce the development of certain stages of *T. cruzi*. The negative effects of *T. cruzi* on the triatomines depend on the *T. cruzi* strain and are particularly evident when the triatomines are stressed. The intestinal immunity of the triatomines responds to ingested blood-stage trypomastigotes of some *T. cruzi* strains and affects many intestinal bacteria, but not all and not the mutualistic symbionts. The specific interaction between *T. cruzi* and the bacteria is evident after the knockdown of antimicrobial peptides: the number of non-symbiotic bacteria increases and the number of *T. cruzi* decreases. In long-term infections, the suppression of intestinal immunity is indicated by the growth of specific microbiota.

## 1. Introduction

The protozoan parasite *Trypanosoma cruzi* (Kinetoplastida, Trypanosomatidae) is the causative agent of Chagas disease [1,2]. This disease, also known as American trypanosomiasis, is endemic in Latin America, i.e., South and Central America, Mexico, and the southern United States of America. There, its insect vectors, the triatomines, are present. Migration has brought the disease to other countries, particularly to other regions of the United States and Canada, but also to some countries in Europe, the eastern Mediterranean, Africa, and the western Pacific [3,4,5]. In countries outside America, no triatomines or other insects are infected with *T. cruzi*, but the parasites can be transmitted through blood transfusions and organ transplants. However, the screening of donors varies greatly between countries [6,7,8]. Also in Germany, blood and organ donors from immigrants from Latin America are not systematically screened [9]. However, *T. cruzi* was found when blood from an immigrant and her daughter was fed to triatomines (Schaub, unpublished). Fifty years ago, the WHO classified Chagas disease as one of the “Big Six” tropical diseases (leprosy, leishmaniasis, trypanosomiasis, malaria, schistosomiasis, and filariasis) and supported investigations within the framework of the “Special Programme for Research and Training in Tropical Diseases” [10]. Today, it is 1 of more than 20 neglected tropical diseases, all characterized by deficiencies in hygiene and public policy, high prevalence in poor populations, difficulties in diagnosis and treatment, and significant economic impact [8].

The fight against Chagas disease is mainly based on insecticide campaigns against the triatomines [11]. Between 1982 and 2010, this led to a sharp decline in the number of infected people from around 20 million to 8 million (summarized by [12]). Since then, however, it has only improved slightly to 6 to 7 million people [2]. Currently, around 30,000 people are infected each year and 10,000 die each year [13]. The new proposed vector control strategies are based on health education programs and control campaigns [14] and not on new methodologies (see Section 6.1). However, re-infestation after the use of insecticides can only be reduced by improvements to houses [15]. The infestation of houses can be detected by screening human sera for antibodies against the vector’s saliva [16]. As resistance to insecticides has developed, much greater efforts are needed, including biological control agents such as entomopathogenic fungi [17].

Only mammals are vertebrate hosts of *T. cruzi*. Parasitemia varies greatly between species, and some of them are mainly unaffected by the parasite [18,19]. Curiously, stages of *T. cruzi* that are characteristic of the triatomines develop in the scent glands of opossums [19]. Mammals, and thus also humans, become infected in various ways, mainly vectorially (summarized by [12]). In this way, the parasites in the feces and/or urine of the vectors penetrate mucous membranes or wounds in the skin. Even a small number of metacyclic trypomastigotes are sufficient to penetrate the skin of mice after fecal deposits on the puncture wound of the mouthparts [20]. Oral infections occur through the consumption of infected meat or drinks made from fruit juices or sugar cane that contain feces or crushed triatomines. In the latter case, the risk is increased by the survival of trypanosomes in dead triatomines [21]. The route of infection has a significant impact on the outcome of the infection [22]. Blood transfusions and organ transplants from infected people as well as congenital transmission from mother to child also lead to infections (summarized by [12]).

After infection of humans, the course of the disease varies from person to person, but can be divided into two phases, the initial acute phase and the chronic phase [23,24,25]. In the acute phase, the flagellate invades all types of host cells that contain a nucleus, as it requires purines from the host for its development. It multiplies intracellularly until the host cell is exhausted and ruptures, and then invades the blood capillaries, where it can usually be easily detected in the blood with a light microscope [26]. Non-specific symptoms such as swelling of the lymph nodes near the infection site and fever occur. The chronic phase begins after about 1–2 months. The parasites are rarely detectable in the blood, but develop in hemocultures or the vectors used for xenodiagnosis [26]. The indeterminate chronic phase is hardly symptomatic and lasts several years to decades [23,24]. In the final chronic phase, organ dysfunction occurs due to the intracellular development and destruction of cells. The heart and intestines enlarge and mega-organs develop. These pathological effects lead to death [24,27].

The development of a vaccine against the parasite is hardly possible, as ethics prohibit experimental infections of humans without the possibility of a consistently successful treatment. For this purpose, only two compounds, benznidazole and nifurtimox, have been available for >50 years (summarized by [12]). They are recommended for the initial acute phase, but often lead to severe side effects. In addition, some *T. cruzi* strains are not affected [28,29,30]. If congenital infections are detected early, treatment is very successful in almost all children (summarized by [7]). New drugs are currently being tested (summarized by [12]), as well as nanoparticles for the oral administration of benznidazole [31].

In the vector, the triatomine insects, *T. cruzi* develops in the intestinal tract and in the excretory Malpighian tubules (summarized by [32]). In addition to flagellates, fungal and bacterial microbiota, including mutualistic symbionts, colonize the gut. Studies on the interactions between *T. cruzi*, triatomines, and the microbiota offer the opportunity to find new ways to interrupt transmission. The present review is based on the most recent review [12] and provides an updated overview of *T. cruzi*, the vectors, and the microbiota in triatomines and their interactions.

## 2. *Trypanosoma cruzi*

*T. cruzi* is a flagellate. All have a kinetoplast as part of a single mitochondrion, subepidermal microtubuli, a surface coat, and a flagellum [23]. The trypanosome multiplies by longitudinal divisions. Rare genetic recombination leads to a predominantly clonal genetic structure of the populations (summarized by [33]). *T. cruzi* strains have been classified by molecular biological methods into six main evolutionary lineages, designated TcI to TcVI and Tcbat (summarized by [33,34]). Often several of them are distributed in a single country (summarized by [35]), e.g., all TcI to TcVI are present in Bolivia, but one is not found in patients and another only in a small group of people [36]. Within each evolutionary lineage, the biological heterogeneity of strains varies greatly [30,37], but the protein profiles of TcI strains correlate with differences in virulence [38]. Many mixed infections with strains from different evolutionary lineages occur in mammals and triatomines [19]. Mixed infections of clones that belong to the same evolutionary lineage can only be detected through labor-intensive cloning of samples from the field and determination of the biological characteristics. Another aspect is the influence of host genetic diversity on disease transmission and severity [39]. Therefore, generalizing the results obtained with a single strain is misleading.

All *T. cruzi* develop four major stages, the non-dividing trypomastigote and the multiplying amastigotes, epimastigotes, and spheromastigotes (summarized by [40]). In mammals, vector-derived metacyclic trypomastigotes invade the host cells and transform into multiplying amastigotes. After the host cell is exhausted, they develop into blood trypomastigotes. These enter the blood capillaries and infect new host cells or circulate in the blood which is ingested by the triatomines. In the vector, they transform into spheromastigotes and epimastigotes and many different intermediate stages. They are heme-auxotrophic and take up heme originating from the digestion of hemoglobin by the insect presumably in the flagellar pocket [41]. Finally, metacyclic trypomastigotes develop [42,43].

## 3. Vectors

**Figure 1:**
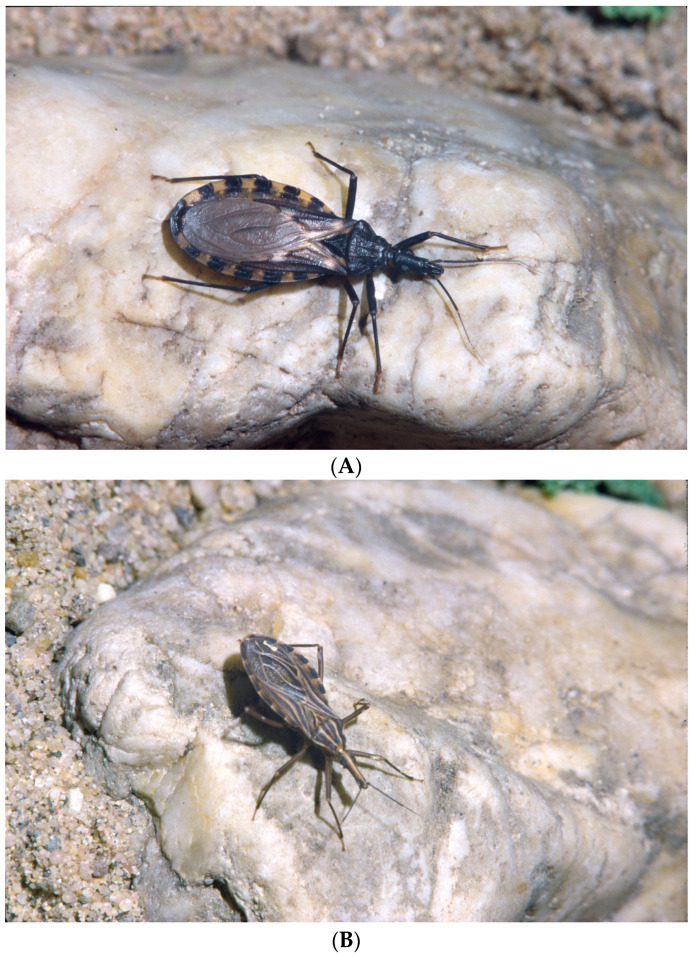
Adults of triatomines. (**A**) Male *Triatoma infestans* (body length: 2.6 cm); (**B**) male *Rhodnius prolixus* (body length: 1.6 cm).

The metacyclic trypomastigotes are transmitted by triatomines which are taxonomically classified to the order Hemiptera, the family Reduviidae (assassin bugs), and the subfamily Triatominae [11]. Around 150 species belong to the Triatominae [44]. *Triatoma rubrofasciata*, the only triatomine species that is distributed worldwide, develops in rat nests in ports of the tropics and subtropics. A few species occur in India, but most are found between the Great Lakes of North America and southern Argentina [11,45,46]. Depending on their habitat, they are divided into sylvatic, peridomestic, and domestic species. Peridomestic triatomines suck the blood of hosts that live in the vicinity of the house, e.g., guinea pigs, chickens, and dogs. Only some species have adapted to house conditions, e.g., *Triatoma infestans* (Figure 1A), *Rhodnius prolixus* (Figure 1B), *Panstrongylus megistus,* and *Triatoma dimidiata* [11]. None of these important domestic species are present in all Latin American countries (summarized by [12,45]).

### 3.1. Development, Attraction, and Blood Ingestion

Triatomines are hemimetabolous insects, and post-embryonic development proceeds through five nymphal stages to the adults [11,45]. All post-embryonic stages feed on blood [47]. Four factors mainly determine the time required for pre-adult development of 5 to 12 months: ambient temperature, availability of hosts, and quality of the blood as well as the supply with mutualistic symbionts. Triatomines feed on all terrestrial vertebrates, mammals, birds, and warm reptiles and amphibians (e.g., [48]). Comparing the effects of feeding on different hosts, *Triatoma pallidipennis* requires a significantly shorter period for pre-adult development and *Triatoma barberi* requires fewer feedings after ingesting blood from mammals than from chickens [49,50]. The blood source—bird or mammal—also significantly influences the morphometry of triatomines (summarized by [51]).

The activity of these nocturnal insects is highest at dawn and dusk [52]. In addition to temperature, exhaled carbon dioxide, skin odor, and visual stimuli also guide the triatomines to the host [52]. There, the proboscis, a sheath that surrounds two pairs of thin mouthparts, is swung forward and pressed against the skin with its tip. Then, the mouthparts are pushed out of the proboscis. While the mandibles cut through the skin, the maxillae penetrate the wound and move within the skin to tap a blood capillary (summarized by [20]). Saliva is pumped into the blood capillary [53]. A probing phase is used to assess food quality and the size of the capillary [54,55]. The rate of blood engorgement is influenced by the host [56]. The nymphs ingest 6 to 12 times their own body weight [11]. Depending on their size, triatomines require 3 to 30 min for complete engorgement, which is then sufficient for development to the next nymphal stage. The nymphs often need to ingest additional amounts of blood in the fifth instar [11].

After molting, the nymphs can take up blood three days later at the earliest, but about three weeks after molting, the number of probings is lower and the amount of ingested blood higher (summarized by [12]). After a complete engorgement, the digestion of fifth-instar nymphs takes more than a month in many species. The subsequent starvation period can last for longer periods, up to a year under optimal humidity conditions. This period varies for the different stages [57,58,59].

### 3.2. The Excretory System and the Fate of Ingested Blood

During blood ingestion, the Malpighian tubules begin to produce urine, which flows through the ampullae at the end of the four Malpighian tubules at the border between the midgut and hindgut. Shortly after excretion begins, the ampullae extend their swollen processes far into the rectal lumen [60,61,62]. They probably absorb ions, water, and other compounds and support a region at the beginning of the rectum, the rectal glands, which differ greatly in the ultrastructure and folding of the cuticle.

The ingested blood passes through the ectodermal foregut, which is lined with cuticle, into the endodermal midgut, which is characterized by microvilli on the lumen side. The first region of the midgut is the short cardia, followed by the highly distensible stomach [11,63]. The third region of the midgut is the small intestine, followed by the ectodermal sac-like rectum [40]. The functions of the intestinal regions are different, especially in the midgut. The cardia protects the mutualistic symbionts (see Section 4.4). In the stomach, blood coagulation is prevented by salivary and stomach-derived anticoagulants. The blood is quickly concentrated by the removal of ions and water [64]. The erythrocytes are lysed and the content has a jelly-like consistency or after feeding on guinea pigs, the hemoglobin crystallizes [65,66]. In the whole midgut, the perimicrovillar membranes develop within hours after feeding, are shed from the microvilli and then degenerate [67]. They cover the contents like the peritrophic membranes in other insects. Within eight days of blood uptake, the stored stomach contents are acidified to a pH of 5.2, followed by an increase to a pH of about 6.5 [68]. Carbohydrates and lipids are digested and absorbed [69]. The activity of aminopeptidases in the stomach contents is higher than in the small intestine [70], possibly digesting leukocytes, plasma proteins, and/or erythrocyte membranes. In a comparison of the two intestinal regions using proteomics, more proteolysis proteins are synthesized in the stomach [71]. These are probably precursors of enzymes that are active in the small intestine, as the digestion of hemoglobin appears to be indicated by a color change from red to brown. According to light and electron microscopy, the functions of the stomach are ion and fluid transport and nutrient storage [72], but the digestion of hemoglobin is discussed [68,73]. Peristaltic movements mix the contents and pass them on to the next region, the small intestine.

There, the contents are liquefied and the hemoglobin is digested or digestion continues, which is indicated by the color change. This region is divided into three parts of similar length by a narrow part in the middle. The anterior half contains more digestive cells than the posterior half and the stomach [74]. Digestion is mainly carried out by two proteases, cathepsins B and L [75]. The activity of cathepsins L and D in the small intestine is higher than in the stomach [70,76]. Finally, the proteins are digested by peptidases, and the aminopeptidases are located between the perimicrovillar membranes [77]. The pH changes after ingestion are similar to those in the stomach, but are somewhat delayed: an initial acidification to pH 5.2 is followed by an increase to around pH 6.5 and pH 5.9 in the posterior part of the small intestine [68]. The residues of digestion are passed into the rectum.

These residues are flushed out after feeding due to the enormous amount of urine. Thereby, the conditions change again [78]. After pH 5.9 in the first dark brown drop of excreta, it rises rapidly to pH 8.4 in the urine and remains at this level for 6 to 24 h after blood ingestion. It then drops to pH 6.2, with individual fluctuations between pH 5.7 and 8.3. The osmolality also changes from about 320 mosmol/kg H_2_O in the first drop to about 410 mosmol/kg H_2_O in the fourth drop [78]. This is also the value in the rectal contents one day after feeding, followed by about 760 mosmol/kg H_2_O two days later. Looking at the change in ion concentration, the rectal contents of unfed insects contain mainly potassium and sulphate, which turns into a sodium chloride solution in the first drops of excreta. Later, the concentrations fluctuate greatly [78]. A change in consistency can also be observed. After flushing out the rectal contents, the urine is clear and then becomes cloudy with yellow-white urate spheres until digestive residues reappear [60,78]. Proteins are hardly present in the urine, but the concentrations are high in the digestive residues stored in the rectum. Nutrients are absorbed before defecation [11].

### 3.3. Immune System of Triatomines

Like all insects, triatomines have an effective cellular and humoral immune system (e.g., [45,79,80,81,82]). It is difficult to assess the importance of immune cells in the hemocel for triatomines, as the liquid diet does not injure the intestinal wall and bacterial penetration through the cuticle can only occur after an attack by a predator. Only some parasites such as *Trypanosoma rangeli* penetrate the intestinal wall, invade the hemocel, and interact with the hemocytes [83]. In the hemocel, there are different types of hemocytes: the phagocytes and others that produce different substances. The ingestion of blood induces the production of antibacterial compounds in the hemocytes, but also in the fat body and in the cells of the small intestine in a systemic reaction [84]. However, the activity of other enzymes for antimicrobial compounds, phenoloxidases, is high in the stomach contents and undetectable in the small intestine [85].

The humoral immunity of triatomines includes many antimicrobial peptides induced by the Toll, IMD (immunodeficiency), JAK/STAT (Janus kinase/signal transducer and activator of transcription), JNK (Jun-N-terminal kinase), and MAPK (mitogen-activated protein kinase) signaling pathways [81,82,86,87,88,89,90]. The pathways react to viruses and many types of bacteria. Non-coding RNAs regulate the immune response during these interactions [91,92]. According to molecular biological data and transcriptomes, several genes encoding at least sixteen antimicrobial compounds of different masses are expressed, some of which are encoded by different genes and exist as multiple isozymes (summarized by [12]). The specific memory that reduces the development of bacteria after a second infection (summarized by [93,94]) has not been studied with bacteria and triatomines. This innate immune priming is active in *Plasmodium* infections of mosquitoes (summarized by [95]). In a comparison of immune memory against two strains of *T. cruzi*, it exists against one strain [96,97], but in one group the mortality rate of 70% within 20 days of infective feeding is surprising.

## 4. The Microbiota of Triatomines

Triatomines normally ingest sterile blood. However, various bacteria, fungi, and viruses are present in the salivary glands, the Malpighian tubules, and especially in the intestinal tract (summarized by [12]). Before and during blood ingestion, the microbiota first come into contact with the antimicrobial peptides in saliva and then with those in the intestinal contents, which are secreted by the cells of the intestinal wall. Lysis by these compounds has not been studied in bacteria derived from triatomines in the field. The microbiota can act as pathogens, commensals, or mutualistic symbionts [98]. Mutualistic and antagonistic interactions of bacteria regulate the composition of the microbiota [99] but also the antimicrobial compounds of the vector.

### 4.1. Infection Routes

The microbiome of Triatominae originates from the environment, the host’s skin, or the host’s blood [100]. Microbes enter the bodies of triatomines in different ways. Some viruses are transmitted transovarially from the female to the offspring (summarized by [101]). Intestinal bacteria and fungi originate from oral infections, e.g., ingestion of water and juices, swallowing air before molting [102], contact of mouthparts with skin before and after blood ingestion, and possibly probing eggshells after hatching (summarized by [12]). Presumably, coprophagy is the most important behavior. Contact with feces is necessary to obtain mutualistic symbionts, but bacteria and fungi present in the environment contaminate the deposited feces.

Coprophagy occurs in all nymphal stages [103], as evidenced by increasing percentages of infections with the coprophagically transmitted homoxenous trypanosomatid *Blastocrithidia triatomae* in groups where the opportunity for coprophagy has been offered since the first nymphal stage. Since this also occurs in the presence of uninfected fifth-instar nymphs, triatomines cannot recognize whether the feces contain symbionts [103]. Coprophagy occurs only after feeding and not during starvation, and only when the nymphs can come into contact with liquid and not dry feces [104]. However, in other studies, *T. infestans* nymphs were not attracted to freshly deposited feces, but rather to volatiles in dry feces within 24 h after feeding [105]. In addition, nymphs leave their shelters to defecate [106]. Therefore, coprophagy requires more detailed studies.

### 4.2. Microbiota of Triatomines

According to previous culture-dependent identifications of the bacteria, many different species colonize the intestinal tract of triatomines (summarized by [107]). Molecular biological methods determined even higher numbers. Approximately 500 bacterial species are present in and on the posterior segments of the abdomen of field-cultured *T. dimidiata* [108,109]. Since the recent reviews on the microbiota of triatomines [12,46,83,110], only a few additional results have been published, which are considered below.

In molecular biological identifications of bacteria from field-derived *Rhodnius pallescens*, *R. prolixus*, *Panstrongylus geniculatus*, *Psammolestes arthuri*, *Triatoma maculata,* and *T. venosa,* over 90% of gut communities belong to the phyla Proteobacteria, Actinobacteria (including Actinomycetales), Bacterioidetes, and Firmicutes [111,112]. In *Triatoma gerstaeckeri* and *T. sanguisuga*, the majority of the microbiota also belong to four taxa, one of which is the Actinomycetales [113]. These bacteria are not found in all triatomines, but are often found with different genera [114]. However, the identification of the species is missing. Actinobacteria dominate in *Triatoma rubida*, *T. lecticularia,* and *T. protracta* and also in field-derived *Rhodnius ecuadoriensis*, and in the latter species, laboratory colonies contain significantly fewer microbes than samples from the field [100,115]. Such differences between *T. sanguisuga* nymphs from the laboratory and adults from the field result in different metabolic functions of the microbiota [116]. Also, *Triatoma rubrofasciata*, which is distributed worldwide, and *T. maculata, T. dimidiata, R. pallescens*, *R. prolixus*, *Eratyrus* ssp., and *P. geniculatus* contain high diversity, including Actinobacteria. Some bacterial families are more common in certain species (summarized by [12]).

### 4.3. Identification of Mutualistic Symbionts

The identification of mutualistic symbionts involves the use of sterile nymphs, infection with individual bacterial species, and the sterile maintenance and feeding of axenic nymphs (details in [110]). Mutual symbionts must establish themselves in the nymphs. Compared to sterile nymphs, the nymphs with the mutualistic symbionts show normal development and reproduction. Because triatomines acquire bacteria from other species grown in the same insectarium and because some of these bacteria are partially symbiotic, identifications of mutualistic symbionts should be based on triatomines from the field (reviewed by [117]).

To date, these symbionts have only been identified from four species of triatomines, but only from two symbionts at the species level [110]. The symbiotic effect of *Rhodococcus rhodnii* in *R. prolixus* has been known for 80 years [118]. Because morphological criteria have been used for identification in the past, all mutualistic symbionts of other triatomines have traditionally been considered *R. rhodnii* (e.g., [119]). Using fifth-instar nymphs and adults from the field, we identified the mutualistic symbiont of three other species of triatomines. The mutualistic symbiont of *T. infestans* is *Rhodococcus triatomae*, previously known as *Nocardia* sp. [120]. It was renamed *R. triatomae* with an almost-complete sequence of the 16S rDNA [121], but without identification as a mutualistic symbiont [122]. The need to determine near-complete sequences points to the difficulty in identification. In *Triatoma sordida* and *P. megistus*, an unnamed *Gordonia rubropertinctus*-like isolate and an unnamed *Rhodococcus equi*-like isolate, respectively, induce normal development [110].

### 4.4. Development of Mutualistic Symbionts/Bacteria in Triatomines

The development of mutualistic symbionts is strongly linked to the cardia, the short first region of the midgut. It has deep sac-like infoldings with narrow channels opening into the lumen [123]. The dense bacterial populations in these infoldings are protected from the complement factors in the ingested blood. After infection of sterile first-instar nymphs of *T. infestans* and *R. prolixus,* with the mutualistic symbionts *R. triatomae* and *R. rhodnii*, respectively, axenic maintenance and sterile feeding, and dissections of fifth-instar nymphs at different times after feeding, the number of colony-forming mutualistic symbionts per nymph is lower one day after feeding than in the cardia and in the stomach of unfed nymphs [120]. In the cardia of both triatomines, they increase rapidly, up to five and seven days after feeding, respectively, and then remain at this level. In the stomach the course is very similar, although the increase is somewhat delayed. Ten days after feeding, a total of 8 × 10^8^ colony-forming units/fifth-instar nymph are present in *R. prolixus* and 1.8 × 10^8^ in *T. infestans*, 95–99% of which are in the cardia and stomach. In both triatomines, around 99.9% of them are digested during passage through the small intestine. The majority of bacteria in the rectum, 90,000 colony-forming units/rectum, are excreted with digestive residues after blood ingestion [120]. The strong difference between the stomach and small intestine is also evident seven days after feeding fifth-instar nymphs of *R. prolixus* possessing approximately 60 × 10^8^ and 2 × 10^8^ colony-forming unit bacteria, respectively [124] (without separate consideration of mutualistic symbionts and other bacteria). In *T. sordida*, too, fewer actinobacteria colonize the small intestine than the stomach, but only genus levels are taken into account in these metagenome determinations [125]. In contrast to these data, in a metagenome shotgun sequencing approach of *R. prolixus*, the relative abundance of Corynebacteria, which includes *Rhodococcus*, is similar in the stomach and small intestine three days after blood ingestion, but lower in the stomach two and seven days after feeding [126]. There are probably many non-symbiotic Corynebacteria present.

After infecting first-instar nymphs of *R. prolixus* with about a million of different *Rhodococcus* species and maintaining them, avoiding infections with other bacteria, the entire intestine of the subsequent nymphal stages contained about 10^6^ genome copies of *R. rhodnii*, but only about 10 of *R. triatomae* and also decreasing concentrations of the other *Rhodococcus* species [127]. With *T. infestans,* exactly the opposite happens, i.e., the mutualistic symbiont of *R. prolixus* does not establish [128].

If we summarize the data on the development of mutualistic symbionts, after feeding there is strong development in the anterior regions of the midgut, cardia, and stomach. There are far fewer mutualistic symbionts in the small intestine and rectum. The sequencing approach highlights the importance of identifying the bacterial species to separate the number of mutualistic symbionts from the number of other bacteria.

### 4.5. Functions of Mutualistic Symbionts/Microbiota

Sterile and aposymbiotic triatomines develop a complex disease syndrome after feeding on guinea pigs, rabbits, or humans. Development is delayed, particularly in the late nymphal stage, and adults rarely develop. In addition, digestion, excretion, and tanning are disturbed, which can partly arise from a reduction in the tracheal system (summarized by [129,130]). After feeding sterile nymphs of *R. prolixus* with blood supplemented with either mutualistic symbionts or B vitamins, development is only slightly delayed and digestion, excretion, and tanning are normal [131]. Functioning as a vitamin B supplier is supported by a metagenome shotgun sequencing approach, as various intestinal bacteria of triatomines have the genes for the synthesis of the various B vitamins [132]. *Bacillus megaterium* has enzymes for the synthesis of these vitamins [133] and colonizes the intestines of field-derived *T. dimidiata* [134].

However, there are arguments against the vitamin B hypothesis. Feeding aposymbiotic *T. infestans* with sterile defibrinated pig blood or on mice and chickens throughout nymphal development avoids aposymbiosis effects that occur after feeding on sheep, cow, and human blood (reviewed by [129]). Unfortunately, the concentration of the various B vitamins in the blood of these animals is unknown. A stronger argument against the vitamin B hypothesis is the normal development of nymphs fed various auxotrophic mutants of *R. rhodnii* that are unable to synthesize certain B-complex vitamins [135]. After feeding on a mixture of blood and all B vitamins, *R. prolixus* nymphs developed more slowly than those infected with the mutualistic symbiont [127,136]. Only *R. rhodnii* and not *R. triatomae*, three other *Rhodococcus* species, *Micrococcus luteus,* and *Escherichia coli* supported the normal growth and reproduction of *R. prolixus* nymphs after feeding with defibrinated rabbit blood [127]. Therefore, these authors concluded that “symbiont B vitamin synthesis is probably a necessary but not sufficient function of gut bacteria”.

Are there other compounds that could be provided by the mutualistic symbionts? Since only Actinomycetales are mutualistic symbionts, these are probably peculiarities of these bacteria. All Actinomycetales belong to the Mycolata taxon, in which the cell walls of the bacteria contain mycolic acids [137]. Another compound can be acylglycerols, esters of glycerol and fatty acids, since the enzymes for degradation are only present in the genome of *Rhodococcus* and not in other intestinal bacteria [126].

Summarizing the data on the functions of mutualistic symbionts, the vitamin B hypothesis requires further investigation. Other compounds are conceivable, e.g., mycolic acids and acylglycerols.

### 4.6. Intestinal Bacteriolysis

Bacteriolysis has also already been described in detail [12] and the essential aspects are presented below. When the lyophilized mutualistic symbiont *R. triatomae* is used as a substrate in turbidity assays, the bacteria are not lysed after incubation with homogenates of the stomach and small intestine of unfed *T. infestans* nymphs and those for up to 50 days after feeding [138]. However, when using the Gram-negative bacterium *E. coli* and the Gram-positive *Micrococcus luteus* as substrates, the extracts from the stomach lead to stronger lysis than those from the small intestine. Both intestinal regions of *R. prolixus* show a similar difference [139], which is also reflected in two antibacterial compounds, the midgut glycosidases of *R. prolixus* and the prophenoloxidase activities of *T*. (*M*.) *pallidipennis* [140,141]. Furthermore, the expression levels of genes encoding lysozymes and defensins are higher in the stomach and cardia than in the small intestine of *Triatoma brasiliensis* and *T. infestans* [142,143,144]. After feeding on a mixture of blood and bacteria, transcripts of lysozyme A, B, defensin C, and prolixicin predominate in the stomach of *R. prolixus*, while mRNAs encoding lysozyme B and prolixicin predominate in the small intestine [139]. The expression rates of prolixicin and three annexins genes in the small intestine of this triatomine are higher than in the stomach [139,145]. In long-term starved *T. infestans*, i.e., 50 days after feeding fifth-instar nymphs, resources are targeted and bacteriolysis of *E. coli* is present in the stomach of adults but is not detectable in the small intestine [138].

In zymographs of stomach and small intestine extracts from unfed *T. infestans* nymphs and those up to 50 days after feeding using nonreducing sodium dodecyl sulfate-polyacrylamide gel electrophoresis, more lysis bands of lyophilized *M. luteus* develop when extracts from the latter region are used. Lytic proteins are mainly present at 15 to 16 kDa, the range of lysozymes, and also at 36 and 40 kDa, but never in the molecular range <14 kDa, the range of most triatomine antimicrobial proteins [138]. Similar data are available for the saliva of *T. infestans*. In a 24 h incubation of these gels in deionized water to detect the bacteriolytic activity occurring under hypotonic conditions, additional lysis bands appear [146]. This indicates the presence of an undissociated complex of an antimicrobial peptide with another protein, e.g., proteases. Protein complexes are found in the saliva of triatomines and other insects (summarized by [12]).

Summarizing the data of bacteriolysis, the bacteriolytic compounds appear to regulate the non-symbiotic bacteria. Mutualistic symbionts are not lysed by extracts from both midgut regions [138]. The strong development of mutualistic symbionts in the stomach and the decrease in the small intestine are contradictory to the high bacteriolytic activities in the stomach and low activities in the small intestine. Presumably, antimicrobial factors and digestive enzymes, which are linked in complexes, must be separated to digest mutualistic symbionts.

## 5. Interactions of Triatomines with *Trypanosoma cruzi*

In the following, I will only consider infections via an infected mammalian host. The difficulties in using isolated blood trypomastigotes and metacyclic trypomastigotes as well as epimastigotes have recently been summarized [12].

### 5.1. Effects of the Vector on Trypanosoma cruzi—Development of the Parasite in the Vector

In general, the long coevolution of parasite and vector includes the development of the flagellate in the triatomines, leading to regional coadaptations of both. However, human migration results in both species being introduced to new locations. There, the introduced triatomines are often less susceptible to the local strains of *T. cruzi* than the local native vectors [147,148,149,150]. The introduced flagellates may not develop in these vectors and are excreted under unfavorable conditions. Therefore, the species/strain of triatomine and the strain of *T. cruzi* are relevant for establishment in the vector (summarized by [12,43,46,83,148,151]).

Field populations of triatomines regularly contain mixed infections with different *T. cruzi* strains, recognizable by their membership in different evolutionary lineages (e.g., [152,153]). These strains also interact, leading to an improvement or reduction in the population density of a strain [154,155]. Therefore, the main decision before studying the interactions of *T. cruzi* and triatomines should be the choice of system. If they are to reflect the natural scenario, the parasite and vector should come from the same locality. Handling *T. cruzi* is relatively simple, with standardized cyclic infections of vectors and mice and long-term storage of aliquots at −80 °C (summarized by [12]). It is more difficult to maintain the vector for many years without changing the genetic structure of the colonies. A large number of triatomines were to be captured in the field and divided into different groups. After several years of maintenance, the colonies should be mixed and the offspring divided into different groups again. This reduces inbreeding effects. As control experiments, the infections with *T. cruzi* can be compared with the old data. Using this method, we have obtained very similar infections for 20 years.

*T. cruzi* develops mainly in the intestinal tract [11,156,157,158]. The Malpighian tubules and their end, the ampullae, appear to be less optimal because they are colonized by a very small number of trypanosomes and not in all nymphs and not in all strains [157,159]. Therefore, the excretory system is rarely considered in infected triatomines. This is even more evident for the cardia. Therefore, the present review focuses on development in the stomach, small intestine, and rectum.

#### 5.1.1. Development of *Trypanosoma cruzi* in the Stomach

After ingesting infectious blood, *T. cruzi* faces conditions that are very different from those of the mammalian host. After temperatures of around 38 °C, pH 7.4 and sufficient glucose as a nutrient in the mammal, the temperature in the stomach changes after a short time to that of the environment in the range of 23 to 30 °C (summarized by [25,160]). The consistency changes quickly and becomes jelly-like or a “serum” with hemoglobin crystals. In *T. infestans*, the pH value is between pH 5.2 and pH 6.5 (summarized by [12]). Glucose is no longer available and *T. cruzi* must use amino acids and lipids derived from the vector’s digestion of the blood. However, the flagellate uses carbohydrates and amino acids without a drastic change in its catabolic enzyme levels [161]. In addition, the ingested blood is mixed with the saliva of the triatomine. This appears to affect *T. cruzi* from two evolutionary lineages differently, but requires more detailed studies of activity in the gut (summarized by [12]).

Initial development appears to depend on the combination of *T. cruzi* strain and vector. In one system, all trypomastigotes in the blood are killed in the stomach within four days of ingestion. In another system, individual trypomastigotes are still present three weeks later (summarized by [12]). At one day p.i. of *Dipetalogaster maxima* and *T.* (*M*.) *pallidipennis* with a *T. cruzi* strain from Guatemala, many trypanosomes are aggregated. Blood trypomastigotes shorten, become more oval, and one day later develop into spheromastigotes and then into oval epimastigote-like forms, but never into slender epimastigotes [162]. The formation of epimastigotes in the stomach is controversial (summarized by [12]). However, they are present there in long-term infections after molting, presumably originating from the small intestine [104]. After the subsequent ingestion of blood from chickens or uninfected mice, these epimastigotes are killed or survive, respectively.

Summarizing the initial development in the stomach, there are strong system-dependent differences in the proportions of trypanosomes killed. Aggregations occur regularly, often of amastigotes and spheromastigotes. In some systems, precursors of epimastigotes develop.

#### 5.1.2. Development of *Trypanosoma cruzi* in the Small Intestine

In the small intestine of the vector, some factors, temperature and pH changes, are similar to those in the stomach. The jelly-like content is liquefied. There, trypanosomes transform into epimastigotes, which multiply quickly [163]. Presumably, the triatomine’s blood digestion provides many nutrients. The vector’s digestive enzymes, proteases, lipases, and carbohydrate digestion enzymes, have no effect on the flagellates. The attachment of epimastigotes to the perimicrovillar membranes is controversial (summarized by [12,83]). Since these membranes are only present temporarily, they cannot play a crucial role in development.

The rapid development of the population is evident in our system, where the vector and *T. cruzi* (TcI) originate from the same village in Chile. One week after infection of second-instar nymphs with 8000 to 10,000 blood trypomastigotes per *T. infestans* nymph, approximately 30,000 parasites/nymphs colonize the small intestine [163]. After three to four week starvation periods, the population is reduced slightly before feeding. After feeding subsequent nymphal instars, flagellate populations increase, resulting in approximately 600,000 parasites per fifth-instar nymph [163].

In established infections of *T. infestans,* there are predominantly epimastigotes and various intermediate stages to spheromastigotes, to metacyclic trypomastigotes via spheromastigotes, and very rarely directly to metacyclic trypomastigotes. Then, after periods of starvation of three to four weeks, more round forms develop [163]. Prolonged starvation of fifth-instar nymphs that were originally infected in the first instar leads to a sharp decline in the population, and 60 days after feeding, no more trypanosomes or only small populations are found [57,164]. Since residues of digested blood are present in the small intestine of long-term-starved dead nymphs, the loss of certain compounds probably leads to the death of *T. cruzi*.

Summarizing the development of *T. cruzi* in the small intestine, blood ingestion supports the development of the population. Epimastigotes in particular, but also spheromastigotes, multiply quickly. Morphologically final metacyclic trypomastigotes are only rarely present. Starvation greatly reduces the number of flagellates.

#### 5.1.3. Development of *Trypanosoma cruzi* in the Rectum

The development of *T. cruzi* in the rectum has also recently been reviewed [12,46,83]. Mainly in the rectum, metacyclic trypomastigotes develop [40]. Their number is higher at a maintenance temperature of 30 °C than at 26 and 28 °C [165]. To highlight an extremely important aspect, in the *T. cruzi*/*T. infestans* system from Chile, the number of trypanosomes increases sharply from one nymphal stage to the next and this part is three-times more populated than the small intestine, finally containing 50% metacyclic trypomastigotes [163]. The reason appears to be the possibility of attachment, and approximately two-thirds of the population attach to the cuticle [166,167]. The flagellum attaches with a small hydrophobic area to the wax layer that covers the entire rectal cuticle [61]. This flagellar region contains specific proteins [168]. In *Leishmania*, deletion of the adhesion proteins prevents colonization of the stomodeal valve [169]. In the flagellum of *T. cruzi*, nanotubules are partially dragged when fluids flow and improve adhesion to the cuticle under shear stress [170]. These filaments, along with hemidesmosome-like material, are also present in the enlargements at the attachment site. A hemidesmosome-like attachment plaque between the tip of the flagellum and the substrate develops in many attached trypanosomatids, e.g., in *Crithidia fasciculata* [171]. A specific region of the rectum of triatomines, the four rectal pads, is extremely densely populated with up to four layers of epimastigotes. Epimastigotes in the upper layers attach to the cuticle with the tip of the elongated flagellum. The reason for the preference for the rectal pads, which have lower folds of the cuticle, is probably the connection to the ampullae of the Malpighian tubules, which just cover these pads outside the rectum. In other insects, water and amino acids are heavily absorbed at the rectal pads (summarized by [172]).

Blood ingestion and starvation also affect the population of *T. cruzi* in the rectum. Urine is already produced during blood ingestion, which sometimes leads to excretion before or shortly after cessation of food intake [173,174,175,176] (see Section 3.2). The time to start defecation is important because the risk of infection is higher when defecation occurs on the host. This varies greatly between species and between developmental stages [177]; (summarized by [83]). Defecation after leaving the host indicates limited vector capacity (e.g., [174]).

The excreta contain the *T. cruzi* population from the rectal lumen and the unattached population from the rectal wall. These are mainly trypomastigotes that cannot attach due to their short, free flagellum and surface coat (summarized by [40]). Furthermore, metacyclogenesis is induced within four hours after blood ingestion, but only in one precursor stage, the epimastigote [159]. The triggering factors are hemolymph proteins of about 17 kDa, which pass into the urine [40,178]. However, other factors could also support this, e.g., specific digestive compounds, since metacyclogenesis in *R. prolixus* is reduced after the knockdown of an α-glucosidase, which also influences heme detoxification and digestion [179]. The drastic changes in pH and osmolalities (see Section 3.2) can also be relevant. Osmotic changes are controlled by *T. cruzi* through various mechanisms [180,181,182].

Blood ingestion after prolonged starvation triggers the development of “giant cells”, a stage of multiple cell division [183]. When present one day after feeding, their proportion increases to 30 to 50% but they disappear completely between five and ten days after feeding. Low numbers have previously been reported for the small intestine [184].

Starvation without subsequent feeding also influences the population density of *T. cruzi* in *T. infestans* and *R. prolixus* [164,185]. In established infections, approximately 300,000 flagellates colonize the rectum 20 and 30 days after feeding of fourth-instar *T. infestans* nymphs [164]. Starvation for 60 and 90 days reduces the population to 100,000 and 1000 trypanosomes/rectum, respectively, the same level present 120 days after feeding. The reduction 90 days after feeding is not due to defecation, as almost all nymphs stop defecating and store rectal contents after a 60-day starvation period. Considering the different rectal regions in detail using scanning electron microscopy, after a starvation period of 16 weeks most of the rectal regions are colonized by *T. cruzi*. After another four weeks, the flagellates are only attached to the rectal pads, but in all nymphs [186]. In addition to the number of trypanosomes, the percentages of the different development stages also change. Before starvation, 2% of the rectal population are spheromastigotes and 1% are intermediate drop-like forms, but forty days later this number is 30% [164,183].

To summarize the development of *T. cruzi* in the rectum, the population is larger than in the small intestine, presumably due to the attachment of the epimastigotes. The unattached trypanosomes are flushed out through urine production, which is triggered by blood ingestion. Very rapid metacyclogenesis of epimastigotes is induced by hemolymph proteins in urine. After nymphs that have been starved for a long period of time ingest blood, “giant cells” form for a few days. During starvation, trypanosomes progressively decrease in number and the populations contain more spheromastigotes.

#### 5.1.4. Parasite Load in the Whole Intestine

Recent parasite burden studies used PCR-based methods and the whole intestine. Field-caught *Triatoma pseudomaculata, T. brasiliensis, Panstrongylus lutzi, T. sordida,* and *Triatoma vitticeps* contained up to 6 × 10^10^ *T. cruzi*/intestine units (median 2.3 × 10^3^) [187]. In the qPCR of *T. brasiliensis*, 3.9 to 7.7 × 10^6^ *T. cruzi* per insect were found and in *Triatoma melanica*, TcII-infections resulted in a lower parasitic load compared to TcI and TcIII [152,153]. The difference between TcI and TcII was also evident after the infection of second-instar nymphs and dissection of fifth-instar nymphs using classical counts in Neubauer hemocytometers, resulting in about 1.7 × 10^6^ and 1.4 × 10^6^, respectively, in the whole intestine [164].

### 5.2. Effects of Trypanosoma cruzi on Triatomines

#### 5.2.1. Effects of *Trypanosoma cruzi* on Nymphs and Adults of Triatomines

Several reviews focus on the effects of *T. cruzi* infection on triatomines (e.g., [12,25,32,46,64,83,150,171,188,189]). Summarizing these reviews and considering recent publications, the pathogenicity of *T. cruzi* for the vector is either low or only apparent when triatomines are exposed to adverse conditions. In the laboratory, this may particularly be due to a lack of supply of mutualistic symbionts which is rarely taken into account in publications. In field populations, suboptimal blood sources and starvation, which affect the development of even uninfected triatomines, could support pathogenic effects [58,59,190,191,192].

Comparing uninfected and infected nymphs in laboratory experiments, some *T*. *cruzi* strains significantly retard the development of *R. prolixus* nymphs and reduce survival, while infections with other strains have no effect [193]. Considering starvation as a stress factor, fourth- and fifth-instar nymphs of *T. infestans* survive statistically significantly shorter times, up to 17% less, after first-instar infection [57]. This is also evident in *T*. (*M*.) *pallidipennis*, but not in *Mepraia spinolai* from the field [141,194]. However, differences in the nutritional status of the field samples must be taken into account. This is lower in infected *M. spinolai* than in uninfected *M. spinolai* [195], perhaps reflecting the differences in the composition of the metabolites in the intestinal tract of uninfected and infected *R*. *prolixus* [196]. When determining the concentrations of free amino acids and those in peptides, the composition of peptides in the rectal contents of *T. infestans* differs from that of uninfected nymphs (Kollien and Schaub, unpublished), which may be due to proteases in the surface coat of the epimastigotes [197]. In *M. spinolai* collected in the field during the warming period, significantly more *T. cruzi*-infected second- and fourth-instar nymphs than uninfected nymphs molted after two feedings in the laboratory [198]. This is not evident after collection during the cooling season and in the first and third nymphal instar. Therefore, it is difficult to find an explanation.

Studies of the effects of *T. cruzi* on adult longevity and fertility also vary. Under optimal conditions, not all strains of *T. cruzi* affect the fitness and reproduction of the vector ([199]; summarized by [83]). In detailed investigations of *T. infestans*, infected adults copulate earlier and more frequently than uninfected specimens, increasing reproductive efficiency and decreasing life expectancy [200].

To summarize the effects of *T. cruzi* on nymphs and adults, these depend on the parasite–vector system and are relevant to starvation endurance.

#### 5.2.2. Effects of *Trypanosoma cruzi* on the Behavior of Triatomines

In the field, the infection has no influence on the feeding profiles of the different nymphal instars of *M. spinolai*, which select different hosts [201]. In domestic and sylvatic populations of *T. dimidiata*, the abundance of some sensilla types on the antennae is affected by infection [202], but relevance to orientation remains to be investigated. Since the effects on triatomine orientation to the host have recently been reviewed [46,83], this will be briefly presented. Often, infected nymphs respond more quickly to human odors or approach the host more quickly than uninfected nymphs [203]. In *T. infestans* nymphs, parasite load correlates significantly with the number of movements and distances during the photophase [204]. These changes should not affect the risk of transmission, as each nymphal instar requires only one complete engorgement. However, the risk of transmission to an insectivorous host is increased because infected nymphs leave the shelter in an arena for longer and are preyed on by mice more often than uninfected nymphs [205]. As recently highlighted, these behavioral effects can be explained by competition between the trypanosomes and the vector, leading to an earlier starvation response. Therefore, infected nymphs of *Triatoma rubrovaria* consume more blood than uninfected ones [206]. A reduction in weight results in an increase in the dispersal ability of infected female *T. dimidiata* (summarized by [46,83]). The significance of higher levels of negative geotaxis and higher levels of aggregation of infected *T. infestans* in males and females, as well as the impaired aggregation of infected *T. pallidipennis*, is due to a lower concentration of attractive compounds in dry feces [207,208], and the importance is difficult to assess.

To summarize the effects of *T. cruzi* on the behavior, these effects appear to be due to competition between the trypanosomes and the insect for compounds in the blood.

#### 5.2.3. Effects of *Trypanosoma cruzi* on Immunity

The ingestion of *T. cruzi* also triggers a systemic reaction outside the intestine by hemocytes, fat bodies, and other organs (summarized by [32,83]). Detoxifying proteins are immediately induced [209]. The activity of a phospholipase involved in the production of nitric oxide and lipid metabolism, as well as the expression of the corresponding genes in the salivary glands of *R. prolixus,* is significantly reduced in the infected group 3 days after feeding, but not later [210]. *T. cruzi* induces the synthesis of nitric oxide [84]. This is an immediate response at one day p.i. to blood trypomastigotes of *T. cruzi* in the stomach of *T. infestans* [211,212].

The triggering factor of antibacterial activity lies in the surface coat, since the ingestion of blood mixed with blood trypomastigotes or their shed surface coat increases antibacterial activity in the small intestine of *T. infestans* nymphs, but not ingestion of trypomastigotes without a surface layer or epimastigotes [213] (details in [83]). Comparing the expression levels of antimicrobial peptide genes, only some of them are upregulated (summarized by [83]). Expression of a lysozyme gene is increased in *R. prolixus* at 7 and 14 days p.i. with blood trypomastigotes [214] and 20 days p.i. with epimastigotes, that of the gene that encodes a defensin in *T. brasiliensis* [144]. When using different *T. cruzi* strains, the immune responses differ (summarized by [215]). While the expression of genes encoding nitric oxide synthase and phenoloxidase is increased after infection (reviewed by [83,215]), the expression of other genes is reduced, e.g., of defensin C in *Rhodnius neglectus* [216]. Since recombinant defensin kills *T. cruzi* in vitro [217], beneficial downregulation is possible.

The effects of *T. cruzi* on immunity can be summarized as follows: surface coat components of ingested blood trypomastigotes induce it. However, this does not seem to affect the flagellate, but appears to be relevant for the interactions with the intestinal microbiota of the triatomine (see Section 6 below).

## 6. Interaction of *Trypanosoma cruzi* and the Microbiota of Triatomines

### 6.1. Effects of the Microbiota on Trypanosoma cruzi

Some publications report direct effects of the microbiota on *T. cruzi*. *Serratia marcescens* lyses epimastigotes (summarized by [83]). However, this depends on the strain of the bacterium [218]. Since co-infections of *S. marcescens* and *T. cruzi* occur (e.g., [219]), the significance in natural infections appears to be low. Also, with another bacterium, *Wolbachia,* co-infections with *T. cruzi* occur in the field [111], and an effect of the bacterium on the trypanosome is unlikely (summarized by [12]).

Genetically transformed mutualistic symbionts have been proposed for use in control campaigns [220,221,222,223,224,225]. These bacteria produce a lepidopteran antibacterial peptide, an antibody fragment, an enzyme disrupting surface glycoconjugates, or dsRNA, all of which kill *T. cruzi* in the intestine. As the release of transformed bacteria into the field is still controversial, no large-scale field studies have been conducted to date.

### 6.2. Indirect Effects of Trypanosoma cruzi via Inducing and Suppressing Vector Immunity

These interactions are described in several reviews (e.g., [12,46,83,86,226,227,228]). Blood trypomastigotes induce an upregulation of the synthesis of antimicrobial compounds in the intestine. This impairs the growth of bacteria and fungi and promotes the growth of trypanosomes. The same effect occurs after feeding antibiotics or an inhibitor of nitric oxide synthase [229,230]. In *R. prolixus*, *T. cruzi* infections increase the transcript levels of defensin C and prolixicin, as well as the antibacterial activity in general and specifically the activity of phenoloxidase, but the synthesis of nitric oxide precursors decreases [124,229]. The activation of immunity by *T. cruzi* significantly reduces the abundance of bacteria after feeding in the midgut, but also changes the composition of bacterial species [126] (see Section 4.2 and Section 4.4). *T. cruzi* infections suppress specific bacteria and promote other bacteria. This is indicated by the *T. cruzi*-induced synthesis of TiAP; recombinant TiAP has a bacteriostatic effect against Gram-negative *E. coli* and not against the Gram-positive *Micrococcus luteus* [231].

Infected *T. dimidiata* from Colombia contain more Kineosporiaceae, but less Brevibacteriaceae, Dermabacteriaceae, and Enterobacteriaceae [48]. In infected *T. infestans* from the field, species of five bacterial genera are overrepresented and four underrepresented [232]. In field populations of *T. sanguisuga*, certain bacteria are significantly associated with main evolutionary lineages: Bacillales with infections with TcI, Aeromonadales with TcIV, and Burkhodeliales and Enterobacteriales with TcII/V [113]. In specimens of *T. gerstaeckeri* and *T. sanguisuga* from the field, there is no association with the main evolutionary lineages TcI and TcIV [114].

The advantage of this induction of immunity lies in the effect against the competition of bacteria and the flagellate. The knockdown of *T*. *cruzi*-induced antimicrobial peptides or suppression of immune responses promotes the growth of more bacteria while reducing the number of flagellates compared to the control group [231,233,234]. The specific effect is evident after the silencing of an antibacterial rhamnose-binding lectin from *R. prolixus*, as transcript levels of bacteria are increased, but *T. cruzi* (TcI) is not affected [235].

In field-caught triatomines, infections correlate with a similar or more diverse species composition of the bacteria [109,111,112,219,236,237]. In samples of *T. gerstaeckeri* and *T. sanguisuga* from the field, more Enterobacterales and *Petrimonas* develop in infected specimens [114]. This and the more diverse species composition may indicate immune suppression due to long-term infections with *T. cruzi*: after feeding a mixture of different bacteria or fungi and blood, high numbers of the microorganisms develop only in long-term-infected nymphs and not in uninfected controls [238].

Summarizing these interactions, *T. cruzi* induces as a short-term response the synthesis of antibacterial compounds that kill certain bacteria that might compete with the trypanosomes. Supporting the growth of other bacteria sometimes depends on the main evolutionary lineage of *T. cruzi*. In long-term infections, *T. cruzi* suppresses intestinal immunity, which could be the reason for the over-representation of some bacteria in triatomines from the field.

### 6.3. Interactions of Trypanosoma cruzi with Mutualistic Symbionts

Only five investigations consider the interactions between the mutualistic symbiont and *T. cruzi*. In *R. prolixus,* which has an established infection with the mutualistic symbiont, more trypanosomes are present at 7 days p.i. than in aposymbiotic nymphs, but fewer *T. cruzi* are present at 21 and 35 days p.i. [239]. The initially stronger development is probably due to support from the mutualistic symbionts: Vitamin B supports the development of the homoxenous flagellate *Blastocrithidia triatomae* in *T. infestans* [240]. After feeding a mixture of blood with cell-culture-derived trypomastigotes and with or without *R. rhodnii* to germ-free first-instar nymphs of *R. prolixus*, the population density of *T. cruzi* is similar in both groups [241]. After a knockdown of the immune component rpRelish, which appears to control the expression of defensin A, the population of *R. rhodnii* in the intestinal regions of *R. prolixus* increases, but the concentrations of *T. cruzi* remain unchanged at 7 and 14 days p.i. [242]. However, the infection of these triatomines with epimastigotes significantly affects the population density of *R. rhodnii* [124]. To investigate the possible effects of trypanosomes on the mutualistic symbionts, the mutualistic symbionts *R. rhodnii* and *R. triatomae* were fed to sterile first-instar nymphs of the respective triatomines, *R. prolixus* and *T. infestans*, followed by axenic maintenance and sterile feedings. After the infection with blood trypomastigotes of *T. cruzi* (TcI) in the fifth-instar and dissections up to 10 days p.i., the population densities of the respective mutualistic symbionts in the cardia, stomach, small intestine, and rectum are similar to those of uninfected nymphs [120].

Summarizing the co-infections of triatomines with *T. cruzi* and the mutualistic symbionts, no long-term support of the trypanosome appears to occur.

## 7. Suggestions for Future Research

The main suggestion is that investigations should reflect the natural scenario using only blood trypomastigotes to infect triatomines. Should old, well-classified strains of *T. cruzi* be used? These strains were cultured in vitro for many years and lost selection by mammalian or vector hosts. There are probably several “Y” strains with different properties. Therefore, better local strains are used. However, strains of *T. cruzi* not only show an enormous variability in development in experimentally infected mammals, but also in the vector [193,243]. Since it is easier to use a strain that develops rapidly in mammals, such strains are preferred. However, in an identification of *T. cruzi* in *T. infestans* from Chile, only one out of five isolates developed into visible parasitemia in mice without immunosuppression by cyclophosphamide [243]. One of these isolates also multiplied somewhat less intensively in the vector [163] (see Section 5.1.4). Before generalizing, more lower-virulence strains should also be used in investigations of the interactions with the vector.

Taking vectors into account, fewer than about 10 of approximately 150 triatomine species are included in studies of the interactions with *T. cruzi* [215]. Sylvatic species in particular are underrepresented. When establishing triatomines in the laboratory, large quantities should be caught in the field, divided into three to four groups of at least 30 individuals each, and reared separately for a few years. After mixing, the genetic variation should be very similar to that of the original population (see Section 5.1). Important open questions for the vectors are the role of cardia, the concentrations of different carbohydrates in the different regions of the intestine, the digestive processes in the stomach, and more details about the rectal conditions. According to preliminary tests with microelectrodes, which cannot be continued, the oxygen concentrations present are very low (Zinkler and Schaub, unpublished).

Investigations of interactions of flagellate and vector should begin with freshly established triatomine stocks from the field, optimally the population used to isolate *T. cruzi*. The following scheme is optimal: After isolation from field-caught triatomines, mammals should be infected to enable simple sterile in vitro cultivation and the flagellates should be cloned. Then, the main evolutionary lineages should be classified to select different lineages. (Without cloning, different subpopulations develop at different times after infection.) After infection of the progeny of triatomines from which *T. cruzi* originates with epimastigotes and the development of metacyclic trypomastigotes in the rectum, aliquots of feces and urine should be mixed with an antifreeze and stored at −80 °C. Thereby, the infection of experimental mammals in subsequent experiments always begins with identical doses of parasites. Using this standardization, many questions can be solved and potentially related to evolutionary lineages, e.g., the development of slender and stout forms of blood trypomastigotes in the vector [244], the killing of blood trypomastigotes in the stomach of the vector, the development of *T. cruzi* in the presence of guinea pig hemoglobin crystals, the relevance of vector-derived compounds in metacyclogenesis, and the attachment compounds at the tip of the flagellum of epimastigotes. Other important open questions are the role of trypanolytic factors in the stomach (see Section 5.1.1) and the time course of activity of salivary compounds in the intestine. Epimastigotes dominate there and secrete cyclophilin, which inactivates the vector-derived salivary trialysin [245].

Much more information is still missing about the mutualistic symbionts and the interaction with and within the intestinal microbiome. Mutualistic symbionts are only known for the following systems: *R. rhodnii* in *R. prolixus*, R. *triatomae* in *T. infestans*, a *Rhodococcus equi*-like isolate in *P. megistus,* and a *Gordonia* sp. in *T. sordida* [110]. Details on the identification process of mutualistic symbionts and modifications were recently published [12,110] and are not repeated here. In addition to identifying more mutualistic symbionts, the compounds that they release to the triatomines should also be identified, e.g., by biochemical determinations of the various B vitamins in mutualistic symbiont-infected nymphs and feeding membrane fractions and the cytosol without contaminating symbionts to sterile nymphs. Vitamin transporters known for mosquitoes should also be looked for [246].

Since the intestinal tract of triatomines is colonized by many bacterial species, interactions between them and with the mutualistic symbionts should be investigated. Do bacteria from the *Bacillus subtilis* group present in the midgut of field-derived *T*. (*M*.) *pallidipennis* produce antimicrobial compounds [180,247] and alter the composition of the microbiota in triatomines? The presence of a bacterium does not necessarily include colonization, since spores may survive in the insect. After feeding sterile nymphs with blood supplemented with different triatomine-derived bacteria, the majority did not colonize the intestine [122]. Since not all triatomines in the field have mutualistic symbionts, other bacteria seem to act symbiotically. Another possibility are optimal blood donor hosts (see Section 4.2 and Section 4.4). The paradoxical situation of high antibacterial activity in the stomach without the lysis of mutualistic symbionts and low antibacterial activity in the small intestine with the lysis of mutualistic symbionts is very interesting. Does lysis occur through complexes of antibacterial factors and digestive enzymes that are activated in the small intestine? (See Section 4.6).

Considering the fascinating interactions of *T. cruzi* and microbiota, the open questions are the identification of the surface compounds of *T. cruzi* that initiate the immune response and the method of the specific modifications by the flagellate. Does *T. cruzi* produce antibacterial compounds or are the effects “only” due to the synthesis of specific antimicrobial compounds of the triatomine? Different evolutionary lineages of *T. cruzi* should be included.

## 8. Conclusions

Will investigations of the interactions between *T. cruzi*, the triatomines, and the microbiota lead to new approaches to prevent the transmission of Chagas disease by triatomines? New methods could discover new aspects of interactions. They should be based on the natural scenario and use optimal systems. Only with such systems can the pathogenic effect of *T. cruzi* on the vector be quantified and the relevance for transmission assessed. The small number of parasite/triatomine systems examined so far makes it difficult to generalize because the genetics of *T. cruzi*, the triatomines, and the microbiota are too diverse. Many questions about the interactions of *T. cruzi*, triatomines, and the microbiota require detailed investigation. The accumulation of metagenomic, proteomic, and metabolomic data will provide new insights into these trypanosome–triatomine–microbiota systems, but the enormous amount of data requires a critical review of the relevance.

## Data Availability

Detailed data are included in the respective publication.
